# Activation and function of receptor tyrosine kinases in human clear cell renal cell carcinomas

**DOI:** 10.1186/s12885-019-6159-2

**Published:** 2019-11-05

**Authors:** Qing Zhang, Jian-He Liu, Jing-Li Liu, Chun-Ting Qi, Lei Yan, Yu Chen, Qiang Yu

**Affiliations:** 1 Shanghai Institute of Materia Medica, Chinese Academy of Sciences, 555 Zuchongzhi Road, Room 2-224, Shanghai, 201203 China; 2 The Department of Urology, Xin Hua Hospital Affiliated to Shanghai Jiao Tong University School of Medicine, 1665 Kongjiang Road, Shanghai, China

**Keywords:** Receptor tyrosine kinases (RTKs), Activation and function, Clear cell renal cell carcinomas (ccRCCs), Targeted therapy, PDGFRβ, Stroma cells

## Abstract

**Background:**

The receptor tyrosine kinases (RTKs) play critical roles in the development of cancers. Clear cell renal cell carcinoma (ccRCC) accounts for 75% of the RCC. The previous studies on the RTKs in ccRCCs mainly focused on their gene expressions. The activation and function of the RTKs in ccRCC have not been fully investigated.

**Methods:**

In the present study, we analyzed the phosphorylation patterns of RTKs in human ccRCC patient samples, human ccRCC and papillary RCC cell lines, and other kidney tumor samples using human phospho-RTK arrays. We further established ccRCC patient-derived xenograft models in nude mice and assessed the effects of RTKIs (RTK Inhibitors) on the growth of these cancer cells. Immunofluorescence staining was used to detect the localization of keratin, vimentin and PDGFRβ in ccRCCs.

**Results:**

We found that the RTK phosphorylation patterns of the ccRCC samples were all very similar, but different from that of the cell lines, other kidney tumor samples, as well as the adjacent normal tissues. 9 RTKs, EGFR1–3, Insulin R, PDGFRβ, VEGFR1, VEGFR2, HGFR and M-CSFR were found to be phosphorylated in the ccRCC samples. The adjacent normal tissues, on the other hand, had predominantly only two of the 4 EGFR family members, EGFR and ErbB4, phosphorylated. What’s more, the RTK phosphorylation pattern of the xenograft, however, was different from that of the primary tissue samples. Treatment of the xenograft nude mice with corresponding RTK inhibitors effectively inhibited the Erk1/2 signaling pathway as well as the growth of the tumors. In addition, histological staining of the cancer samples revealed that most of the PDGFRβ expressing cells were localized in the vimentin-positive periepithelial stroma.

**Conclusions:**

Overall, we have identified a set of RTKs that are characteristically phosphorylated in ccRCCs. The phosphorylation of RTKs in ccRCCs were determined by the growing environments. These phosphorylated/activated RTKs will guide targeting drugs development of more effective therapies in ccRCCs. The synergistical inhibition of RTKIs combination on the ccRCC suggest a novel strategy to use a combination of RTKIs to treat ccRCCs.

## Background

Kidney cancers are common in developed countries and are notoriously difficult to be treated. Ninety percent of kidney cancers are renal cell carcinomas (RCCs) which originate from tubular structures of the kidney. They are subdivided into clear cell carcinoma (ccRCC), papillary carcinoma, chromophobe, and oncocytoma. The remaining 10% are transitional cell carcinomas, which are derived from cells lining the renal pelvis and ureter [1, 2]. Standard treatments for RCCs are surgery (partial or total nephrectomy) for localized kidney cancer, targeted therapies and immunotherapies for metastasized cancer. Seventy-five percent of the RCCs are ccRCCs which are poorly sensitive to traditional chemotherapy. Targeted therapies are also limited by the lack of knowledge of genetic mutations in the ccRCC cells.

The receptor tyrosine kinases (RTKs) are a large family of transmembrane receptors with 58 members in human [3]. The ligand-induced dimerization of the RTKs lead to phosphorylation/activation of the receptors as well as the downstream signaling molecules [4, 5]. RTKs play critical roles in the development of many diseases, especially cancer. Dysregulations of the RTK signaling through point mutation, gene amplification, overexpression, chromosomal alterations, and/or constitutive activation are key factors in oncogenesis [4, 6–11]. However, the activation and function of the RTKs in ccRCC have not been fully investigated.

Previous studies in ccRCCs have mainly focused on RTKs gene expressions [12, 13]. No genetic mutations of RTKs have been reported in the ccRCCs. The only molecular mechanism related to RTKs in ccRCCs is dysregulation of the pVHL/HIF axis [14, 15], which drives expression of VEGF and PDGFβ and, hence, activation of their receptors VEGFR2 and PDGFRβ [16–20]. Therefore, current treatments for ccRCCs are mostly anti-angiogenic tyrosine-kinase inhibitors (TKIs) targeting VEGFR, which include pazopanib, sunitinib, axitinib, sorafenib, and bevacizumab [21, 22].

In the present study, we analyzed the phosphorylation/activation/ patterns of RTKs in 10 ccRCC patient samples, 4 RCC cell lines, and 4 other kidney tumor samples. Our data revealed that multiple RTKs were activated in the ccRCCs and the phosphorylation patterns of the RTKs in the ccRCC patients were similar to each other but different from adjacent normal tissues and the other kidney tumors. Treatments with a combination of RTK inhibitors based on their phosphorylation patterns in the ccRCC-derived xenografts effectively inhibited the cancer cell growth. These data suggest an effective therapeutic strategy to treat ccRCC patients.

## Methods

### Collection of primary kidney tumors

The renal tissue specimens were collected in compliance with local ethics regulations at the Department of Urology, Xin Hua Hospital Affiliated to Shanghai Jiao Tong University School of Medicine, China. The 10 ccRCC patients were five males and five females (Table 1). The mean age at diagnosis was 65 ± 9. The patient information of 3 other kidney cancer samples and 1 benign renal tumor sample are in Table 2. After surgical resection, tissue samples were lysed in lysis buffer (R&D Sytems, AYR001B) for protein lysates on the ice or fixed in neutral buffered formalin (10%) for histology staining, or immediately processed to establish ccRCC patient-derived xenograft models in nude mice.

**Table 1:** Patient information of renal cell carcinoma (RCC)

No.	Age	Histopathology	Stage
RE0370	72	Clear cell RCC	II
RE0380	56	Clear cell RCC	I~II
RE0390	73	Clear cell RCC	II
RE0400	77	Clear cell RCC	II
RE0410	67	Clear cell RCC	II~III
RE0440	66	Clear cell RCC	II
RE0450	53	Clear cell RCC	I
RE0480	54	Clear cell RCC	II
RE0490	56	Clear cell RCC	II
RE0510	77	Clear cell RCC	II

**Table 2:** Patient information of the other kidney cancers and a benign renal tumor

No.	Age	Histopathology
RE0020	59	Papillary RCC
RE0150	55	Oncocytoma
RE0210	52	Renal pelvic carcinoma
RE0500	52	Cystic nephroma

### Cell lines

786–0(CRL-1932), A-498(HTB-44), ACHN(CRL-1611), and Caki-1(HTB-46) cell lines were obtained from ATCC. 786–0 and Caki-1 cell lines were derived from human primary ccRCC. A-498 and ACHN cell lines were derived from human primary papillary RCCs. 786–0 and ACHN cells were cultured in RPMI 1640 Medium (Gibco) with 10% FBS (Gibco). A498 cells were cultured in Dulbecco’s Modification of Eagle’s Medium (Gibco) with 10% FBS. Caki-1 cells were cultured in McCoy’s 5A Medium (Sigma) with 10% FBS.

### HE staining

Fixed tissues were dehydrated using grades of ethanol (70, 80, 90, 95, and 100%). Dehydration was followed by clearing the samples in two changes of xylene. The samples were then impregnated with two changes of molten paraffin wax, embedded, and blocked out. The tissue sections (7 μm) were stained with hematoxylin-eosin by standard procedures. Stained sections were observed and photographs were taken using a Leica microscope.

### RTK phosphorylation/activation profiling

Human phospho-RTK arrays (R&D Systems, AYR001B) were used according to the manufacturer’s instructions. Briefly, a total of 5 mg protein lysates of in vitro cultured cells, or 10 mg protein lysates of clinical samples and mouse xenografts were diluted in the kit-specific dilution buffer and incubated with blocked membranes overnight. The membranes were washed and incubated with anti-phospho-tyrosine-HRP antibody for 2 h. The membranes were washed and exposed to chemiluminescent reagent. The arrays were photographed using Image Station 4000MM PRO system (Carestream). The pixel densities of various spots were collected and quantified with its software. The average signal (pixel density) of the pair of duplicate spots was determined for each RTK. A signal from the PBS negative control spots was used as a background value. And signals of reference spots in the corners were used for normalization among different arrays. Phospho-RTK relative value was calculated according to the following formula: Phospho-RTKx relative value = (INTx-INTnc)/(INTref-INTnc). INTx is the pixel density of RTKx, INTnc is the pixel density of background,and INTref is the density of reference spots.

### Western blotting

Proteins were separated by SDS-PAGE and transferred to a nitrocellulose membrane. The membrane was blocked in TBS containing 0.1% Tween 20 (TBST) and 5% nonfat milk for 1 h at room temperature and then incubated overnight in TBST containing 5% bovine serum albumin and primary antibodies. Membranes were then washed with TBST and incubated with horseradish peroxidase-conjugated secondary antibody for 1 h, and immune complexes were detected by immobilon Western chemiluminescent HRP substrate (WBKLS0500, Millipore). Primary antibodies are anti-phospho-EGFR (#3777), anti-EGFR (#4267), anti-phospho-PDGFRβ (#3161), anti-PDGFRβ (#3169), anti-phospho-InsulinRβ (#3024), anti-InsulinRβ (#3025), anti-phospho-VEGFR2 (#2474), anti-VEGFR2 (#9698), anti-phospho-Met (#3077), anti-Met (#3148), anti-phospho-Akt (#4060), anti-phospho-Erk1/2 (#4370). All antibodies were purchased from Cell Signaling Technology. The membranes were photographed using Azure Biosystems (c300) and were quantified using its software (Analysis Toolbox). The density ratio of interest proteins to GAPDH or β-Actin were calculated.

### Xenograft models and treatment

The female BALB/c nude (nu/nu) mice were purchased from Beijing Vital River Laboratory Animal Technology Co., Ltd. and used for implantation at the age of 6–8 weeks. They were maintained under specific pathogen-free conditions, and food and water were supplied ad libitum. Housing and all procedures were performed according to protocols approved by the Ethics Committee of Shanghai institute of materia medica. Subcutaneous xenografts were established by injection of 5× 10^6^ cells or one piece (2mm^3^) tumor per mouse to right flank. Tumor formation was monitored each week. Each subcutaneous tumor was measured using a caliper, and tumor volumes were calculated as follows: 0.5× length× width^2^. Nude mice with ccRCC patient-derived xenografts of approximately 100 mm^3^ were allocated randomly into 4 experimental groups and orally treated with 3 mg/kg/d Crizotinib (*n* = 6), 30 mg/kg/d Lapatinib (n = 6), combination of Crizotinib and Lapatinib(n = 6), or vehicle (n = 6) for 21 days. Mice were euthanized in a CO_2_ chamber for 2 h after the last treatment. Crizotinib and Lapatinib were purchased from Selleck Chemicals.

### Immunofluorescence staining

Cryosections were blocked in PBS containing 5% normal donkey serum for 2 h at room temperature. Sections were incubated over night at 4 °C with the primary antibodies against PDGFRβ (ab32570, rabbit Anti-PDGF Receptor beta antibody, 1:50, Abcam), Pan-Keratin (#4545, mouse anti-pan-keratin antibody,1:50, CST), Vimentin (sc-7557, goat anti-vimentin antibody, 1:50, Santa Cruz). After washed with PBS three times, the sections were incubated for 1 h at room temperature with Alexa Fluor 594-labeled donkey anti-rabbit IgG (A21207,1:400, Invitrogen), Alexa Fluor 488-labeled donkey anti-mouse IgG (A21202,1:400, Invitrogen) and Alexa Fluor 555-labeled rabbit anti-goat IgG (A21431,1:400, Invitrogen). Sections were washed three times in PBS, followed by mounting tissue with Dako fluorescence mounting medium. Photographs were taken using a Leica DMi8.

### Statistical analysis

Data were represented as mean ± SEM. T test was used in human phospho-RTK studies. Two-way ANOVA with Tukey post hoc test was used in mouse xenograft treatment studies. Statistical significance was established for *P* < 0.05, *P* < 0.01, and *P* < 0.001.

## Results

### Pathological examination of the ccRCCs and their adjacent tissues

**Fig. 1:**
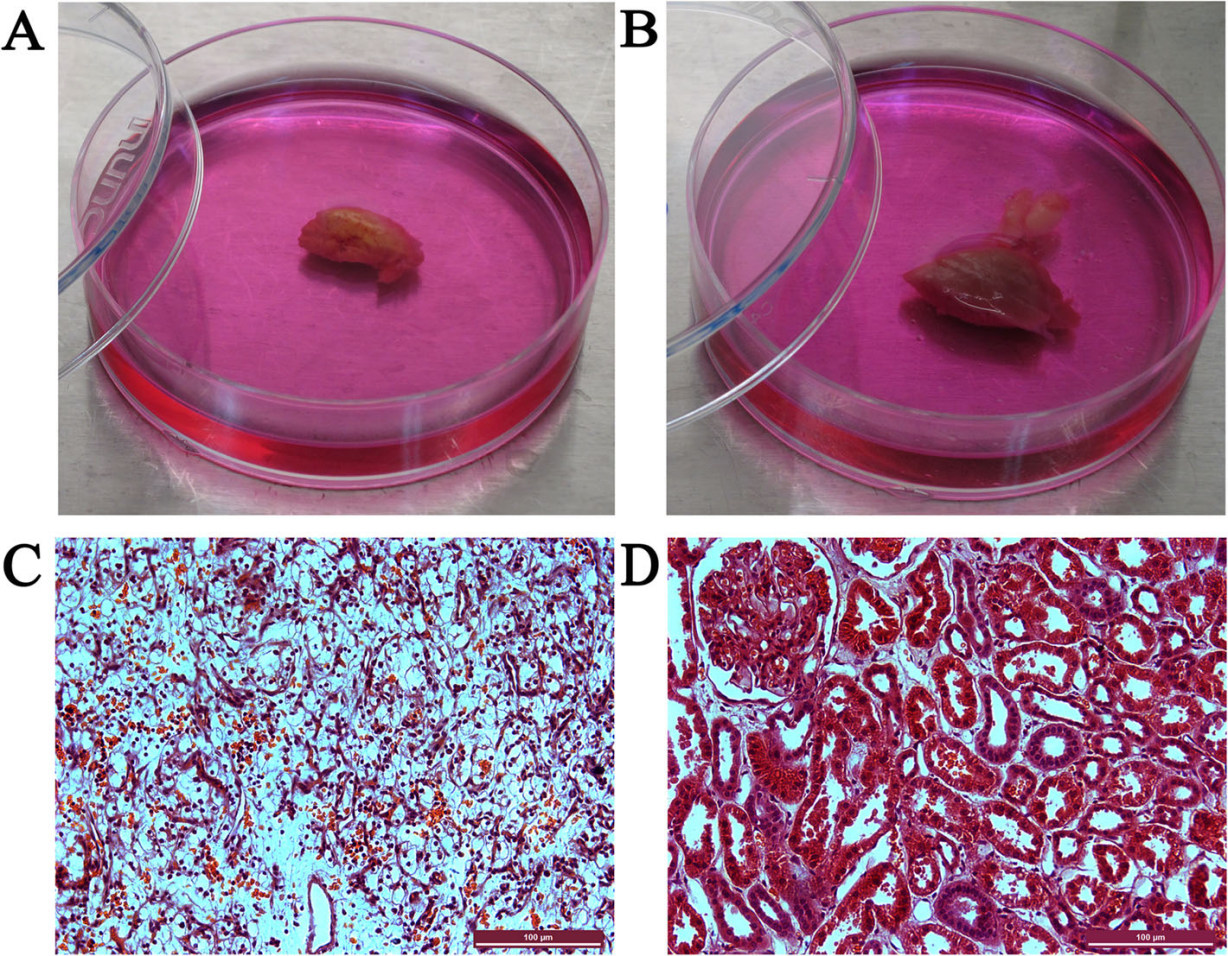
A gross presentation and HE staining of a representative ccRCC total nephrectomy sample and its adjacent tissue. **a**. A typical gross presentation of ccRCC with a bright yellow color. **b**. The adjacent normal tissue. **c**. HE staining of a section of the ccRCC with transparent empty cytoplasm and well-defined cell borders. **d**. HE staining of a section of the adjacent tissue with normal glomerulus, proximal convoluted tubules, and distal convoluted tubules. Scale bars represent 100 μm

To examine the histopathology of the kidney tumors, HE staining was performed. Gross examination of the resected tumor samples revealed that the ccRCCs were all bright yellow in color, due to their intracellular lipid accumulation (Fig. 1a). In contrast, the adjacent normal tissues of the ccRCCs showed normal flesh color (Fig. 1b). In HE staining sections, the ccRCC cells showed transparent and empty (water clear) cytoplasm with well-defined cell borders (Fig. 1c). The nuclei of ccRCCs were round. Architecturally, the ccRCCs displayed compact-alveolar or acinar growth patterns. The small nests were surrounded by a well-developed network of thin-walled vessels. An abundance of extravasated red blood cells were observed in the tumors. The glomerulus, proximal convoluted tubules, and distal convoluted tubules in the cortex of the kidney could be seen in adjacent tissues (Fig. 1d).

### The phosphorylation patterns of the RTKs in the ccRCC patient-derived tumors were similar

**Fig. 2:**
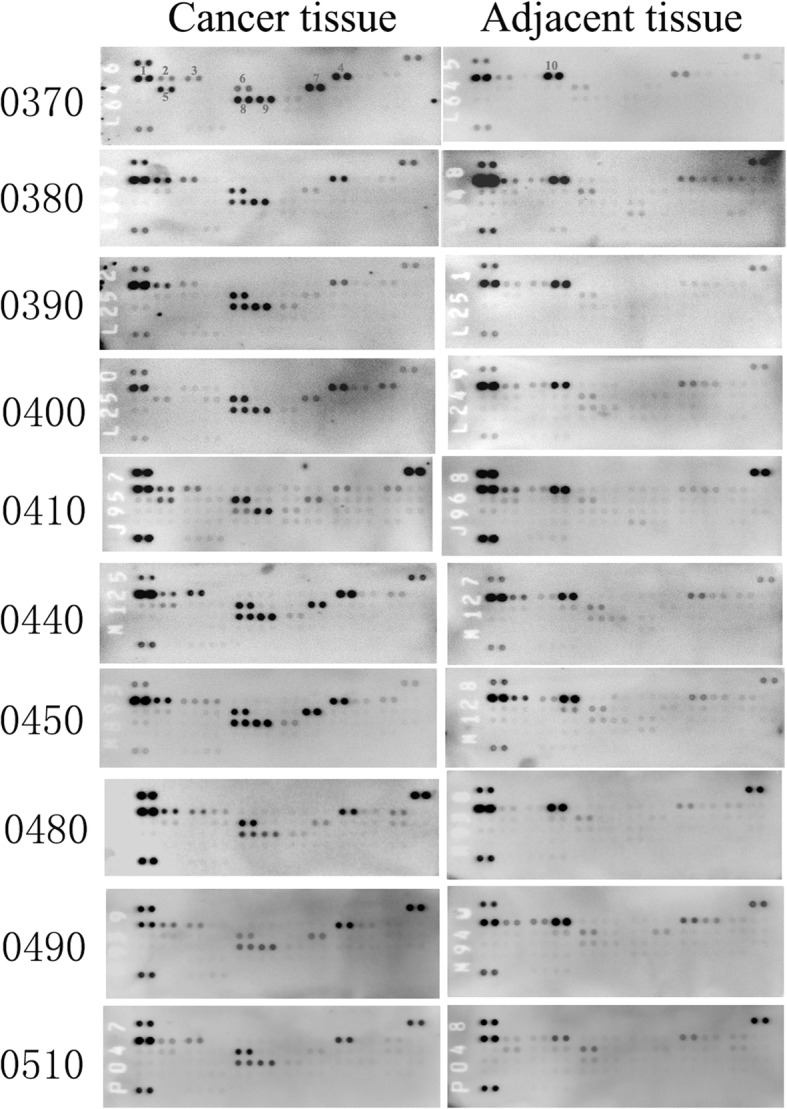
Patterns of phospho-RTK in 10 pairs of human ccRCCs and adjacent tissues. Each RTK was in duplicate. Positive control spots are located on the top left, top right, and bottom left of each array. (1. EGFR; 2. ErbB2; 3. ErbB3; 4. Insulin R; 5. HGFR (Met); 6. PDGFRβ; 7. M-CSFR; 8. VEGFR1; 9. VEGFR2; 10. ErbB4)

**Fig. 3:**
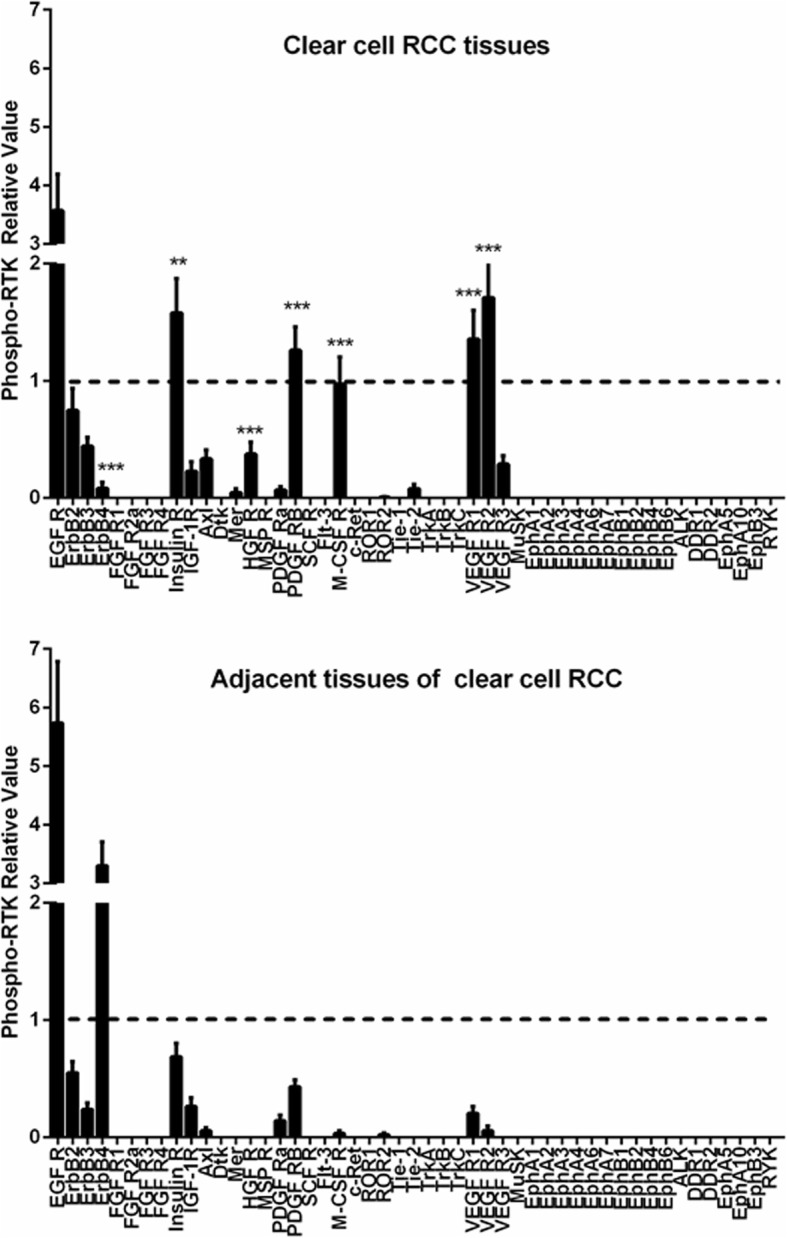
The relative levels of the phospho-RTKs in human ccRCCs and adjacent tissues. The phospho-RTK levels were measured using the human phospho-RTK array kit. *P* < 0.05 (*), *P* < 0.01 (**), and *P* < 0.001(***) vs. adjacent tissues of clear cell RCC. Data were represented as mean ± SEM

**Fig. 4:**
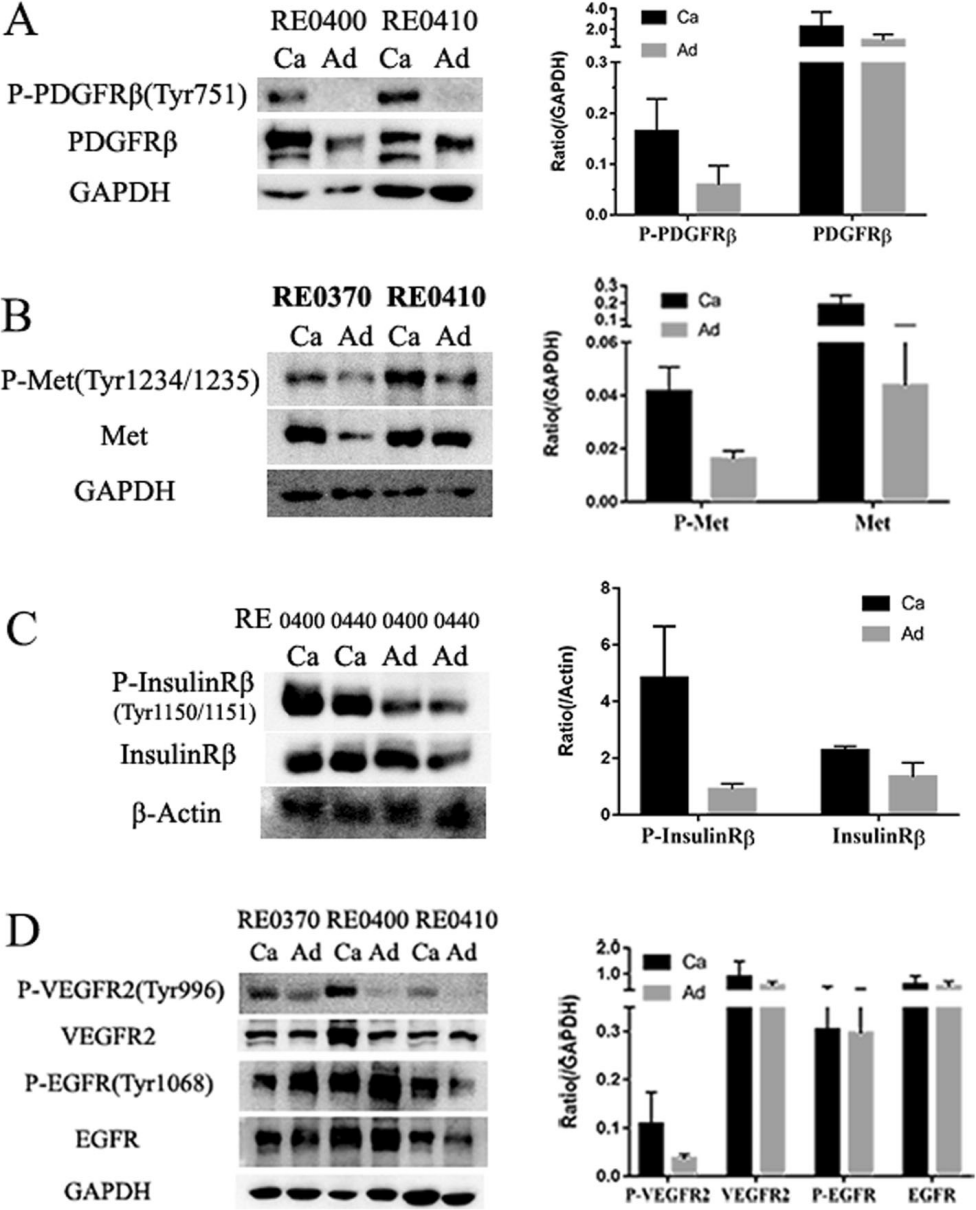
Western blotting analyses of the tissue lysates of the human ccRCCs (Ca) and adjacent tissues (Ad). Tissues were lysed and protein was analyzed by Western blotting using antibodies as indicated. GAPDH and β-Actin antibodies were used as loading controls

To understand the expression and phosphorylation of the RTKs in the ccRCCs, we analyzed 10 pairs of primary ccRCCs and their adjacent non-tumor kidney tissues using human phospho-RTK arrays which evaluate the relative phosphorylation levels of 49 receptor tyrosine kinases (Additional file 1: Fig. S1). 9 RTKs (EGFR1–3, Insulin R, PDGFRβ, VEGFR1, VEGFR2, HGFR, and M-CSFR) were found to be phosphorylated in the ccRCC samples (Fig. 2 and Fig. 3). Comparing to their adjacent normal tissues, Insulin R, HGFR, PDGFRβ, M-CSFR, VEGFR1, and VEGFR2 were specific to the ccRCCs. Among them, the phosphorylation levels of Insulin R, PDGFRβ, VEGFR1, and VEGFR2 were significantly increased in all the ccRCC samples. The phosphorylation levels of HGFR (spot #5) and M-CSFR (spot #7) varied among the samples. HGFR was highly phosphorylated in RE0370 and RE0410 samples while M-CSFR was highly phosphorylated in RE0370, RE0440, and RE0450 samples. This RTKs activation patterns of ccRCCs were different from that of their paired adjacent tissues in which only the EGFR family members, particularly EGFR and ErbB4, were significantly phosphorylated. These findings were further verified by Western blotting analyses. The phosphorylation levels of Insulin Rβ (Tyr1150/1151), PDGFRβ (Tyr751), VEGFR2 (Tyr996), and HGFR (Met Tyr1234/1235) were found to be increased in the tumor tissues in comparison to the paired adjacent tissues (Fig. 4). In addition, the protein levels of some of the RTKs (Insulin Rβ, PDGFRβ, VEGFR2, or Met) were also increased in certain tumors. The protein expression patterns of PDGFRβ and VEGFR2 in tumors were also different from their adjacent tissues (Fig. 4a, d).

### The RTK phosphorylation patterns of ccRCC patient-derived tumors were different from that of human ccRCC cell lines, papillary RCC cell lines, and other kidney tumor samples

**Fig. 5:**
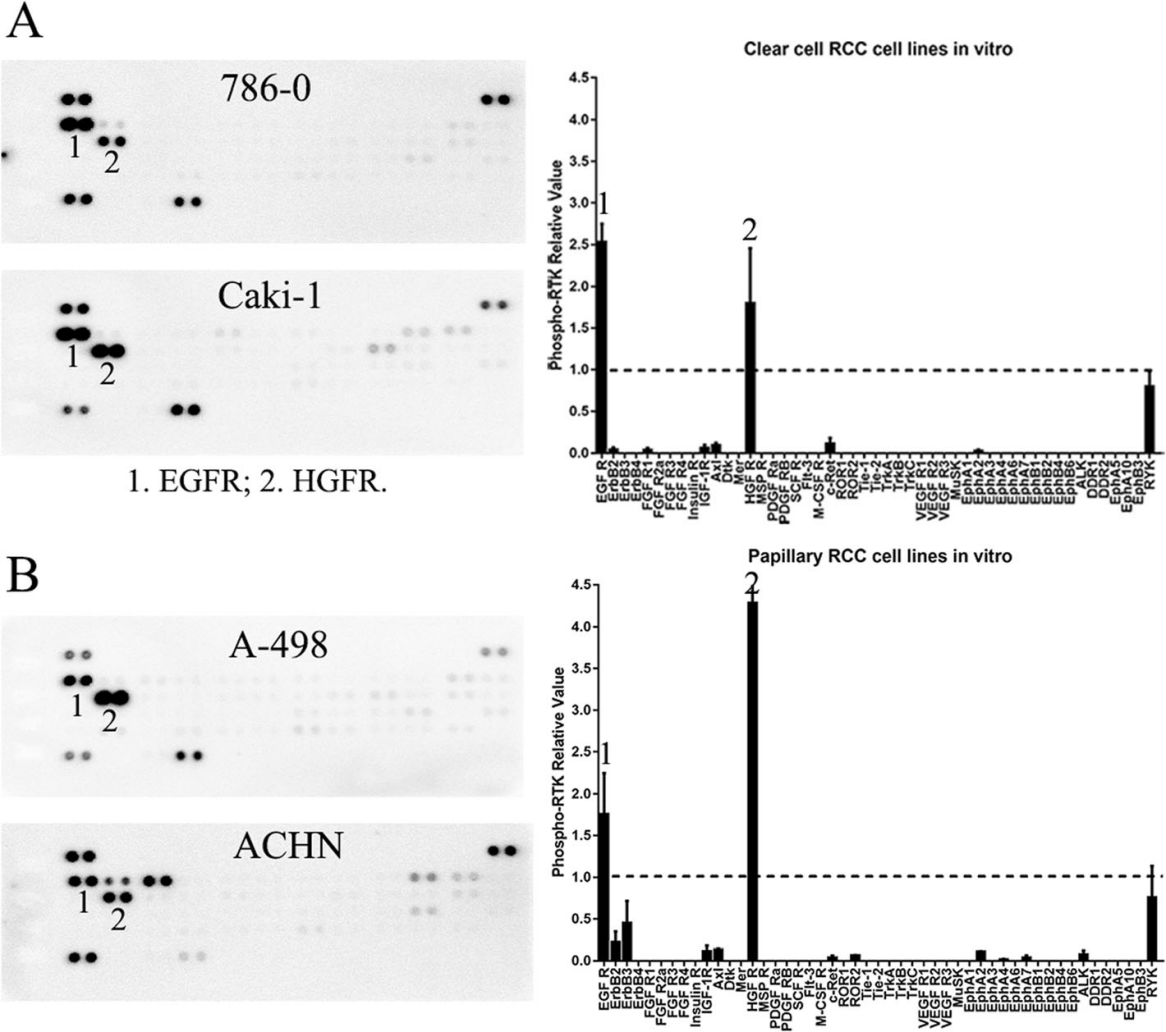
Patterns of the phospho-RTKs in the human ccRCC (**a**) and papillary RCC (**b**) cell lines. EGFR (1) and HGFR (2) were all activated in the four RCC cell lines

**Fig. 6:**
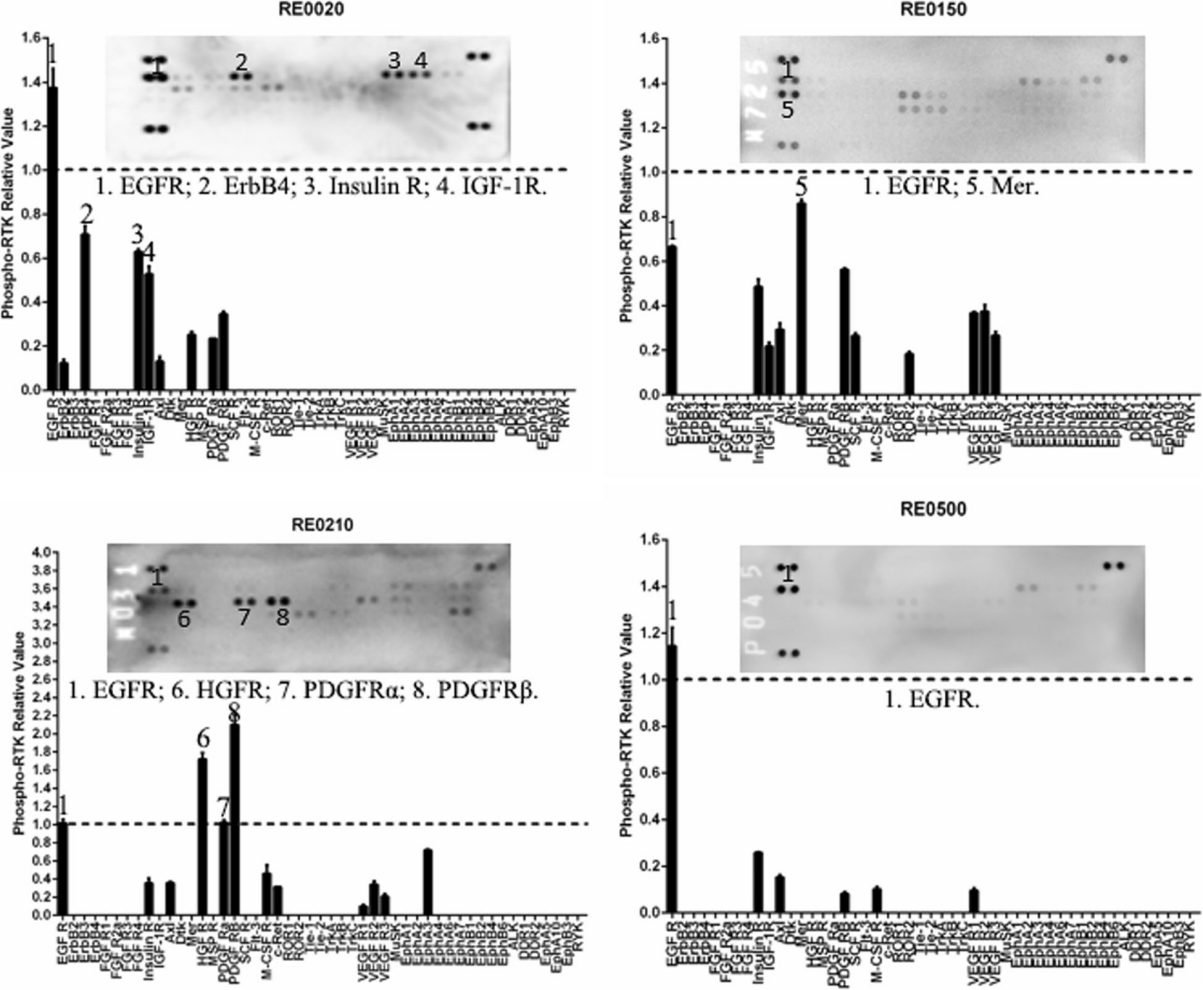
Patterns of phospho-RTKs in the other kidney cancer samples and the benign renal tumor. The relative levels of the phospho-RTKs were calculated and presented under each array blot

To determine whether the RTK phosphorylation patterns in the ccRCCs are specific, we evaluated the RTK phosphorylation patterns in 2 ccRCC cell lines, 2 papillary RCC cell lines and 4 other types of kidney tumor samples. The RTK phosphorylation patterns of the four human RCC cell lines were similar with each other (Fig. 5). The EGFR family and HGFR were highly phosphorylated in all the four cell lines. In contrast, the RTK phosphorylation patterns of the four other types of tumor samples, namely a papillary RCC (RE0020), an oncocytoma (RE0150), a renal pelvic carcinoma (RE0210), and a cystic nephroma (RE0500), were different from each other and were also different from that of the ccRCCs, except EGFR, which was highly phosphorylated in all samples (Fig.6). ErbB4, Insulin R, and IGF-1R were phosphorylated in the papillary RCC (RE0020), Mer (Axl family) was phosphorylated in the oncocytoma (RE0150), and HGFR, PDGFRα, and PDGFRβ were phosphorylated in the renal pelvic carcinoma (RE0210, Fig.6). In the benign renal tumor, namely the cystic nephroma sample (RE0500), only EGFR was phosphorylated (Fig.6). These data demonstrated that the RTK phosphorylation patterns of the ccRCCs were specific.

### The RTK phosphorylation pattern of the ccRCC sample in the xenograft was different from that of the primary samples

**Fig. 7:**
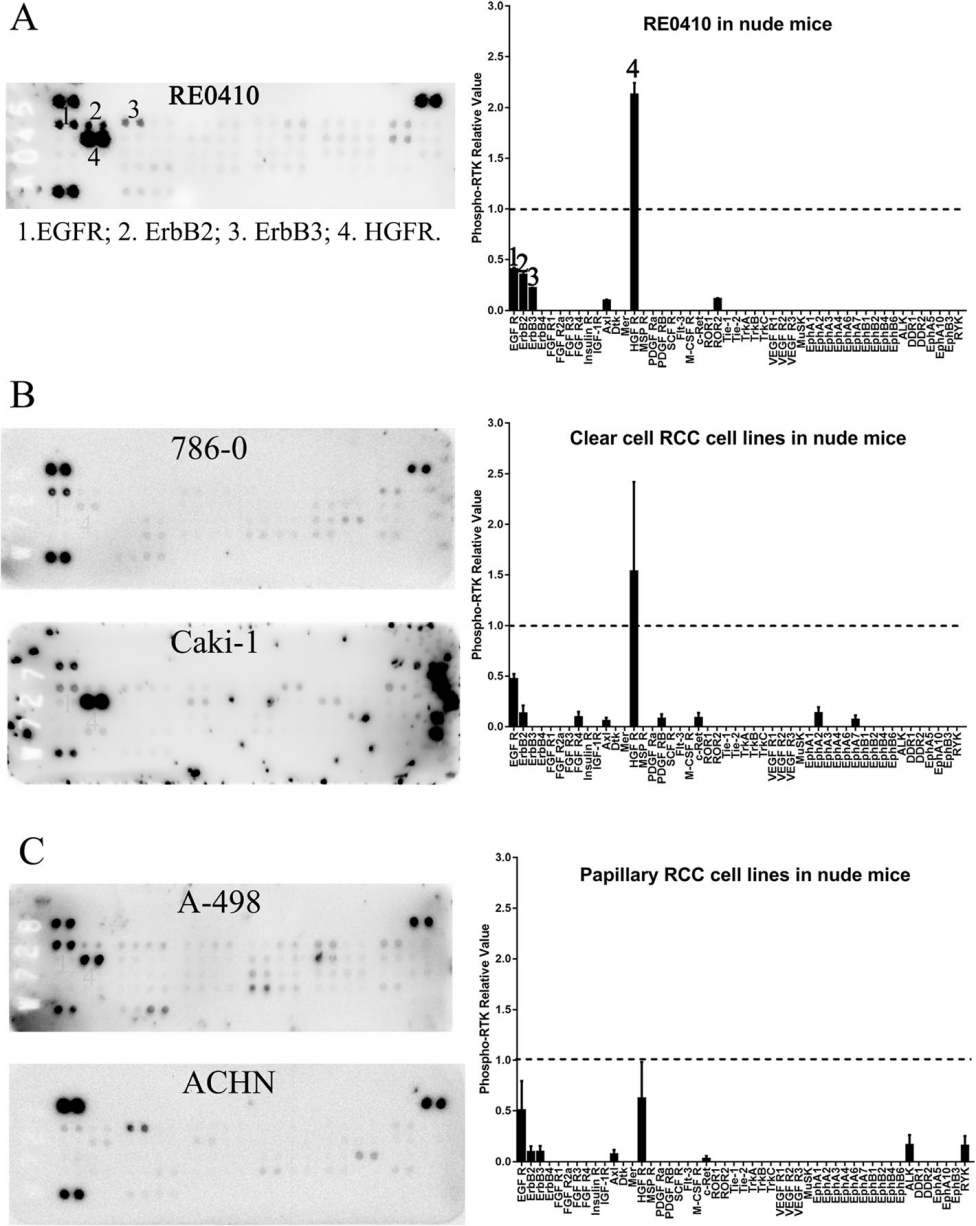
Patterns and quantitation of the phospho-RTKs in the xenograft mice of 1 patient-derived ccRCC sample (RE0410, **a**), 2 human ccRCC (**b**) and 2 papillary RCC (**c**) cell lines

In order to treat the tumors with tyrosine kinase inhibitors based on their RTK phosphorylation patterns, we tried to establish tumor xenograft models using the patient-derived tumor samples as well as the cancer cell lines. Thirty-five tissue pieces from the 10 samples of the ccRCCs were subcutaneously implanted into 35 nude mice. Only one xenograft (RE0410) grew successfully. We then analyzed the RTK phosphorylation pattern of this ccRCC explant. The RTK phosphorylation pattern of the xenograft was different from its original primary sample (RE0410). Only the phosphorylation of EGFR family (EGFR, ErbB2 and ErbB3) and HGFR were maintained at high levels while that of the other RTKs decreased (Fig.7a). In contrast to the poor tumorigenicity of the ccRCC samples from patients, the established cell lines of ccRCC and papillary RCC were highly tumorigenic. Both EGFR and HGFR remained phosphorylated in all four of the cell line-derived xenograft samples, although their phosphorylation levels decreased in vivo (Fig.7b, c). These data demonstrated that the RTK phosphorylation patterns in the xenografts changed and the success rate of subcutaneous grafting of ccRCC samples was low in nude mice.

### Combination of TKIs synergistically inhibited the growth of ccRCCs in vivo

**Fig. 8:**
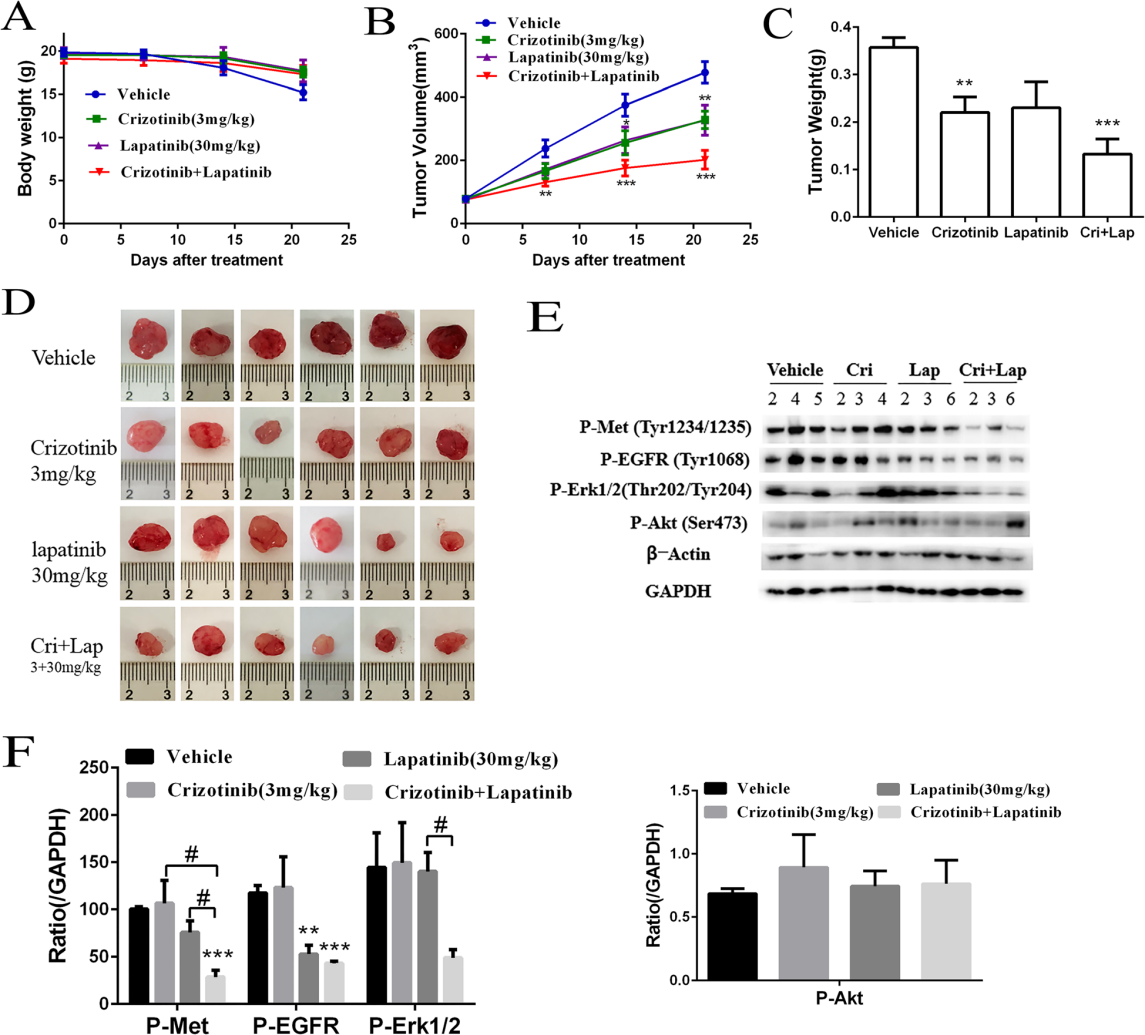
Combination of TKIs synergistically inhibited human ccRCC growth in vivo. **a** and **b**. The body weights and tumor volumes during the drug treatment. The ccRCC xenograft nude mice were treated with vehicle, crizotinib (Cri), lapatinib (Lap), or combination of them for 21 days. Tumors were excised and photographed at the end of treatments. **c**. The tumor weights at the end of treatment. D. Tumors from ccRCC xenograft nude mice. **e**. Western blotting analyses of P-Met, P-EGFR, P-Erk1/2 and P-Akt levels of the tumors. The numbers underneath the groups represent the serial number of mice. Tumor lysates were processed for Western blot analyses and probed with the indicated antibodies. **f**. The ratios of protein phosphorylation levels relative to GAPDH. *P* < 0.05 (*), *P* < 0.01 (**), and *P* < 0.001(***) vs. vehicle group. Drug combination group was compared with the crizotinib group or lapatinib group (*P* < 0.05, #). Data were represented as mean ± SEM

Phospho-RTK array of the ccRCC explants from the xenograft mice showed that three of the EGFR family members and the HGFR were highly phosphorylated in the xenograft tumors. We therefore used the RTK inhibitors targeting EGFR family and HGFR to treat the ccRCC xenograft nude mice. As shown in Fig. 8a, the change of body weight in treatment groups was similar with that in vehicle group. The EGFR inhibitor lapatinib or the HGFR inhibitor crizotinib alone slightly inhibited the tumor growth (Fig.8b). In comparison, the combination of the two inhibitors was much more efficient than the single treatment to inhibit the tumor growth (Fig. 8b). The average inhibition rate of crizotinib, lapatinib, or a combination of them on the ccRCC were 38.24 ± 22.40%, 35.43 ± 37.15%, and 62.79 ± 21.95% respectively (Fig. 8c, d).

To understand the effects of the combination treatment at the molecular level, we examined the effects of crizotinib, lapatinib, or the combination of them on the phosphorylation/activation of their target proteins and their downstream signaling molecules Erk1/2 and Akt. As shown in Fig. 8e and f, the combination treatment synergistically inhibited the phosphorylation of Met, EGFR, and Erk1/2. These data suggested that a combination treatment of the RTK inhibitors based on the RTK phosphorylation patterns synergistically inhibited the RTK-mediated signaling and the tumor growth.

### PDGFRβ was expressed in the periepithelial stroma cells

**Fig. 9:**
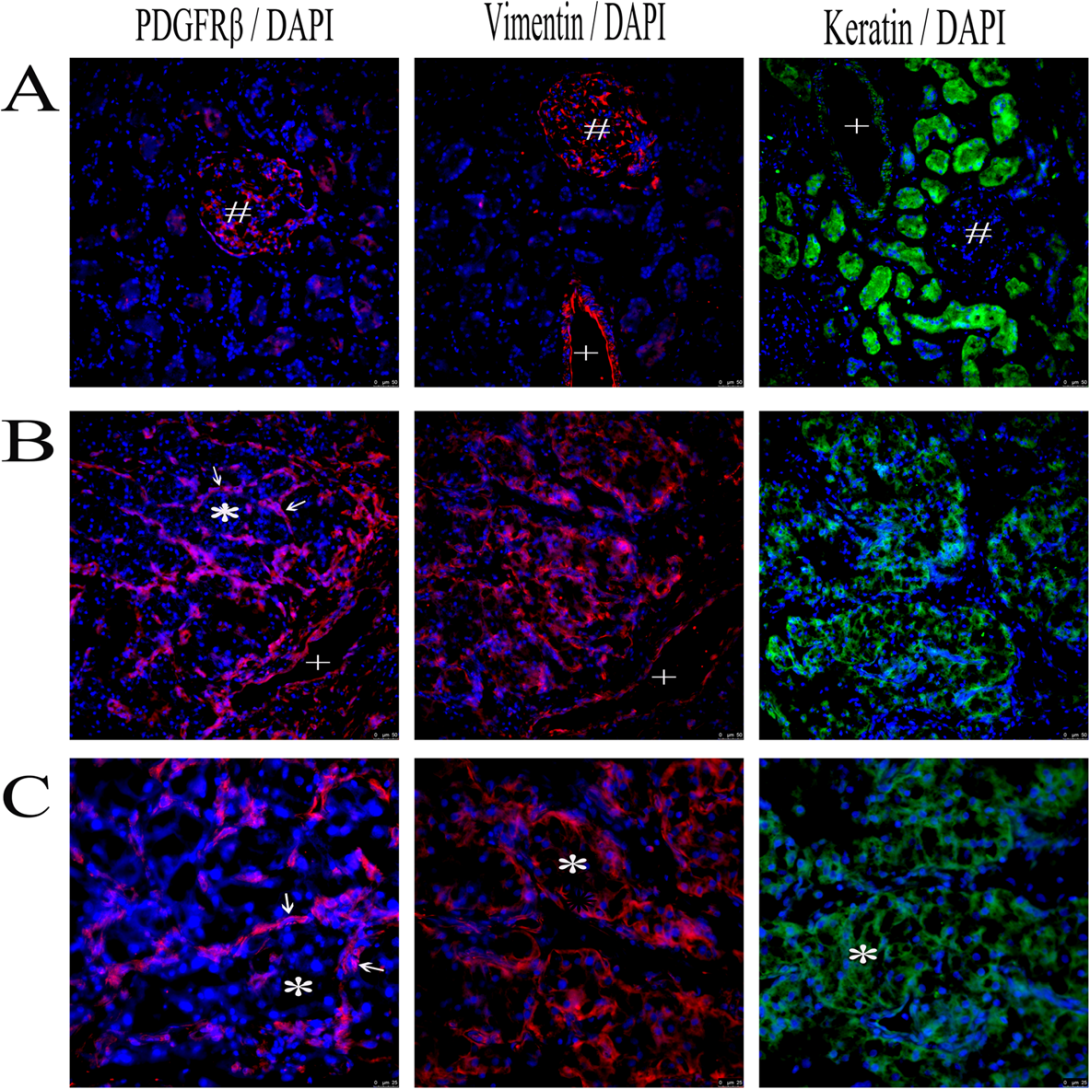
Immunostaining for PDGFRβ (red), Vimentin (red) and Keratin (green) in a pair of human ccRCC tissues. Cell nucleus was stained blue by DAPI. **a**. Human ccRCC adjacent tissue (scale bars = 50 μm). **b**. Human ccRCC tissue (scale bars = 50 μm). **c**. Human ccRCC tissue (scale bars = 25 μm). Arrows indicate PDGFRβ positive cells surrounding the tumor islands (*) in the ccRCC tissue. # indicates glomerulus and + indicates blood vessel

PDGFRs are usually expressed in stroma cells. To understand the function of the PDGFRβ in the ccRCCs, we analyzed the expression of PDGFRβ in the patient-derived ccRCCs and their adjacent tissues. The PDGFRβ was mainly expressed in glomerulus in the tumor adjacent tissues (Fig. 9a). In the ccRCC tumor tissues, PDGFRβ was present in the vimentin-positive stroma cells surrounding the tumor islands and blood vessels (Fig. 9b, c). But the keratin-positive epithelial cells were mainly localized in the tumor islands which were PDGFRβ-negative (Fig. 9b, c). These results suggest that the PDGFRβ expressing cells were periepithelial stroma cells in the ccRCCs.

## Discussion

We identified 9 RTKs that were significantly phosphorylated in the primary ccRCC samples and 6 of which, Insulin R, HGFR, PDGFRβ, M-CSFR, VEGFR1, and VEGFR2, were specific to the ccRCCs samples comparing to their adjacent normal tissues. More importantly, the phosphorylation patterns of the RTKs in the ccRCC patient samples were similar among each other. It is therefore possible that the activation of the 6 ccRCCs-specific RTKs are important for the formation and growth of the ccRCCs. Our data are consistent with previous studies on the expressions and roles of RTKs in ccRCCs. There were several reports demonstrated VEGF/VEGFR activation and HGFR upregulation in patients with ccRCCs [12, 17–20, 23, 24]. The M-CSFR activation we observed in the ccRCC samples may be due to increases and activations of the tumor-associated macrophages in ccRCCs [25–27]. The role of Insulin R in ccRCCs is unclear [28]. There was a report showing that the expressions of Insulin R were similar in ccRCCs and their adjacent normal tissues, but the phosphorylation of the Insulin R was not analyzed in this report [29]. Our data demonstrated that the Insulin R was significantly phosphorylated in the ccRCC samples, but not in the adjacent normal tissues, suggesting that Insulin R may have a role in promoting ccRCC cell growth. However, it was also reported that Insulin R expression correlated with a lower Fuhrman nuclear grade and better patient prognosis [29]. Further studies are needed to clarify the roles of Insulin R in ccRCCs. None the less, these data suggest that the 6 specifically activated RTKs in the ccRCCs may be important targets for the treatment of ccRCCs.

Among the 6 specifically activated RTKs, HGFR and Insulin R were reported to be mainly expressed in the ccRCC epithelial cells [23, 24, 29]. The M-CSF R seems to be expressed in the tumor-associated macrophages [25–27] while the VEGFRs were likely expressed in the blood vessel endothelial cells. The PDGFRβ was found to be mainly expressed within the periepithelial stroma in the ccRCC samples in our study. Similar expression patterns of PDGFRβ were found in breast, prostate, pancreatic, gastric, and oral squamous cell carcinoma cancer cells [30–32]. More importantly, high PDGFRβ expression in fibroblast-rich stroma is commonly associated with poor prognosis [33, 34]. These data suggest that the RTKs in the ccRCC stroma cells may also be abnormally activated to support the growth of the cancer cells. Thus, targeting the activated RTKs in both the cancer epithelial cells and the surrounding stroma cells that associated with poor prognosis may be a primary choice for treating the ccRCC patients.

It is unclear what caused the activation of the RTKs in the ccRCCs. The behavior of the ccRCCs in the xenograft mice, however, indicated that majority of the 9 RTKs might be activated by their corresponding growth factors in the tumor environments. When the cancer cells were implanted into a new environment in the xenograft mice, most of the cancer cells failed to grow, likely because of lack of necessary growth factors to activate the RTKs. The only ccRCC sample that did grow in the xenograft mouse had different RTK phosphorylation patterns from that of the original sample. In addition, the four cancer cell lines, when implanted into the xenograft mice, also showed similar RTK phosphorylation patterns as the primary cancer sample, but different from that of the in vitro growing cells. All these data suggest that the RTK phosphorylation patterns of the cancer cells are not cell autonomous, but rather are determined by their growing environments.

Although we could not reproduce the same RTK phosphorylation patterns of the ccRCC primary cancer samples in the xenograft models, the treatment of the tumor cells in the xenograft mice with a combination of the RTKIs, based on the RTK phosphorylation patterns, successfully inhibited the tumor cell growth, suggesting that the RTK phosphorylation pattern-guided treatment of cancers is an effective therapeutic strategy.

## Conclusions

In summary, we have identified a set of RTKs that are characteristically phosphorylated in ccRCCs. The phosphorylation of the RTKs and the growth of the ccRCCs were determined by the growing environments of the ccRCCs. Treatment of the ccRCC xenograft mouse with a combination of RTKIs based on the RTK phosphorylation pattern of the ccRCC in the new environment synergistically inhibited the growth of the ccRCC. These data suggest a novel strategy to use a combination of RTKIs to treat ccRCCs.

## Additional file

**Additional file 1: Figure S1.** Schematic illustration of the RTK array from the R&D Systems. (TIF 2291 kb)

## Data Availability

The datasets used and/or analyzed during the current study are available from the corresponding author on reasonable request.

## Abbreviations

ccRCCs: Clear cell renal cell carcinomas
EGFR: Epidermal growth factor receptor
HGFR: Hepatocyte growth factor receptor
IGF-1R: Insulin-like growth factor 1 receptor
M-CSFR: Macrophage colony-stimulating factor receptor
PDGFR: Platelet-derived growth factor receptor
RTKIs: Receptor tyrosine kinase inhibitors
RTKs: Receptor tyrosine kinases
VEGFR: Vascular endothelial growth factor receptor
