# Supplementary figures and images for: Activation and function of receptor tyrosine kinases in human clear cell renal cell carcinomas
Source: BMC Cancer. 2019 Nov 5;19:1044. doi: 10.1186/s12885-019-6159-2 (PMC6833303; doi:10.1186/s12885-019-6159-2)

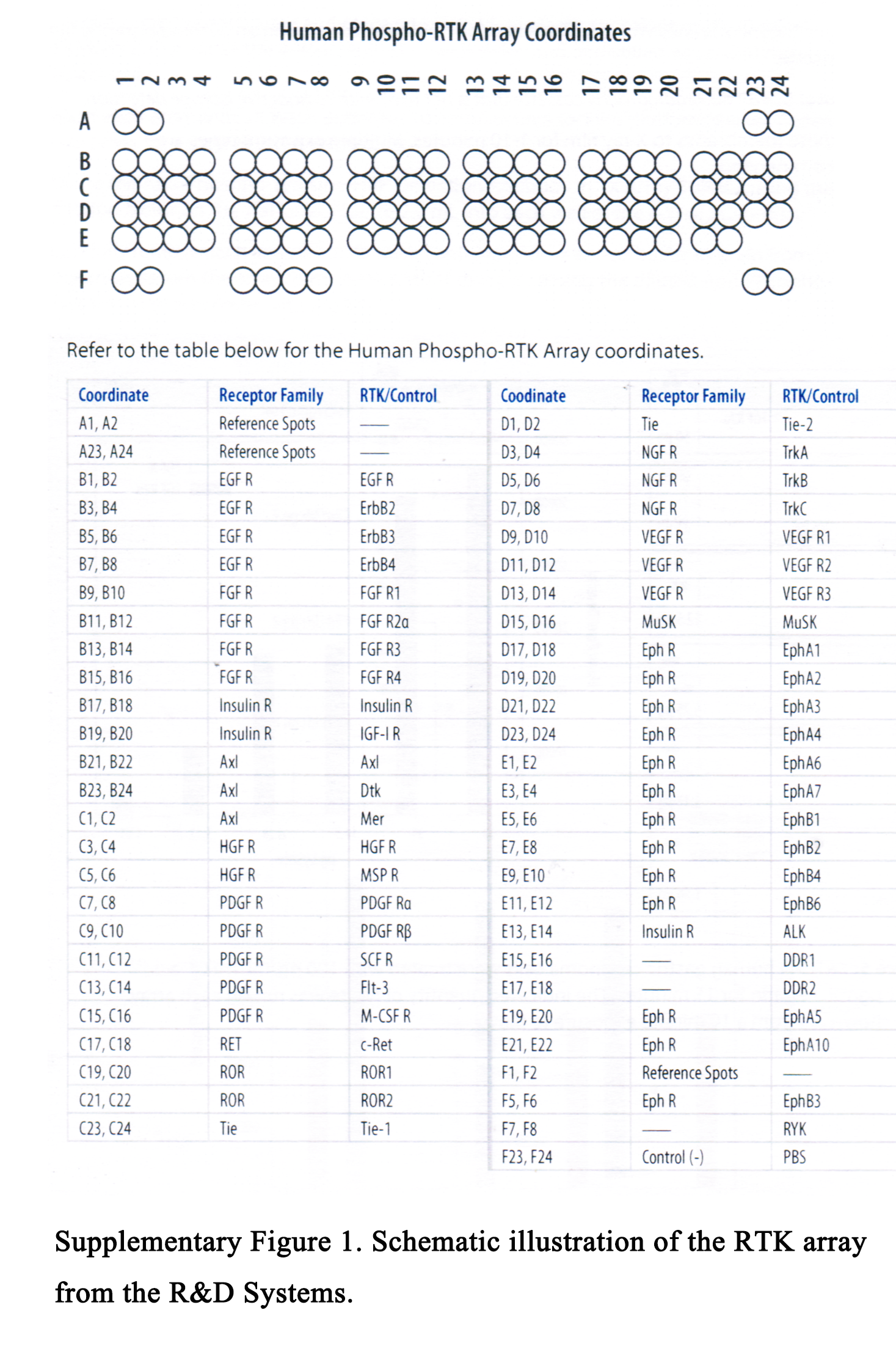

Supplement: Supplementary file 1 — Additional file 1: Figure S1. Schematic illustration of the RTK array from the R&D Systems. (TIF 2291 kb) [file 12885_2019_6159_MOESM1_ESM.tif]
